# Treatment of spasticity in children and adolescents with cerebral palsy in Northern Europe: a CP-North registry study

**DOI:** 10.1186/s12883-021-02289-3

**Published:** 2021-07-12

**Authors:** Gunnar Hägglund, Sandra Julsen Hollung, Matti Ahonen, Guro L. Andersen, Guðbjörg Eggertsdóttir, Mark S. Gaston, Reidun Jahnsen, Ira Jeglinsky-Kankainen, Kirsten Nordbye-Nielsen, Ilaria Tresoldi, Ann I. Alriksson-Schmidt

**Affiliations:** 1 Department of Clinical Sciences Lund, Orthopedics, Lund University, Skåne University Hospital, 221 85 Lund, Sweden; 2 Norwegian Quality and Surveillance Registry for Cerebral Palsy, Vestfold Hospital Trust, Tønsberg, Norway; 3 Department of Pediatric Orthopedics and Traumatology, Children’s Hospital, Helsinki University Hospital, University of Helsinki, Helsinki, Finland; 4 SLF´S Rehabilitation Center- Æfingastöðin, Reykjavík, Iceland; 5 Cerebral Palsy Integrated Pathway, Scotland. Royal Hospital for Sick Children, Edinburgh, UK; 6 Norwegian Quality and Surveillance Registry for Cerebral Palsy, Oslo University Hospital and University of Oslo, Oslo, Norway; 7 Department of Health and Welfare, Arcada University of Applied Sciences, Helsinki, Finland; 8 Department of Children’s Orthopedics, Danish Cerebral Palsy Follow-Up Program, Central Region Denmark, Danish Paediatric Orthopaedic Research Group, Aarhus University, Aarhus, Denmark; 9 Department of Children’s Orthopedics, Danish Paediatric Orthopaedic Research Group, Aarhus University, Aarhus University Hospital, Aarhus, Denmark

**Keywords:** Spasticity, Cerebral palsy, Treatment, Baclofen, Selective dorsal rhizotomy, Botulinum toxin

## Abstract

**Background:**

Spasticity is present in more than 80% of the population with cerebral palsy (CP). The aim of this study was to describe and compare the use of three spasticity reducing methods; Botulinum toxin-A therapy (BTX-A), Selective dorsal rhizotomy (SDR) and Intrathecal baclofen therapy (ITB) among children and adolescents with CP in six northern European countries.

**Methods:**

This registry-based study included population-based data in children and adolescents with CP born 2002 to 2017 and recorded in the follow-up programs for CP in Sweden, Norway, Denmark, Iceland and Scotland, and a defined cohort in Finland.

**Results:**

A total of 8,817 individuals were included. The proportion of individuals treated with SDR and ITB was significantly different between the countries. SDR treatment ranged from 0% ( Finland and Iceland) to 3.4% (Scotland) and ITB treatment from 2.2% (Sweden) to 3.7% (Denmark and Scotland). BTX-A treatment in the lower extremities reported 2017–2018 ranged from 8.6% in Denmark to 20% in Norway (*p* < 0.01). Mean age for undergoing SDR ranged from 4.5 years in Norway to 7.3 years in Denmark (*p* < 0.01). Mean age at ITB surgery ranged from 6.3 years in Norway to 10.1 years in Finland (*p* < 0.01). Mean age for BTX-A treatment ranged from 7.1 years in Denmark to 10.3 years in Iceland (*p* < 0.01). Treatment with SDR was most common in Gross Motor Function Classification System (GMFCS) level III, ITB in level V, and BTX-A in level I. The most common muscle treated with BTX-A was the calf muscle, with the highest proportion in GMFCS level I. BTX-A treatment of hamstring and hip muscles was most common in GMFCS levels IV-V in all countries.

**Conclusion:**

There were statistically significant differences between countries regarding the proportion of children and adolescents with CP treated with the three spasticity reducing methods, mean age for treatment and treatment related to GMFCS level. This is likely due to differences in the availability of these treatment methods and/or differences in preferences of treatment methods among professionals and possibly patients across countries.

## Background

Spasticity is one of the most common manifestations of cerebral palsy (CP). The spastic CP subtype, with spasticity as the dominant motor symptom, represents 78–88% of the population with CP [1]. Spasticity is also present in about 70% of children with dyskinetic CP [2].

Spasticity may cause limited range of active joint motion, with reduced gross and fine motor function and pain [3]. However, spasticity can also improve motor function by compensating for muscle weakness [4]. There are several methods available to reduce spasticity including: botulinum toxin-A therapy (BTX-A), selective dorsal rhizotomy (SDR) and intrathecal baclofen therapy (ITB).

BTX-A produces a dose-related temporary tone reduction of the muscle injected by inhibiting the release of acetylcholine from the motor endplates [5]. BTX-A treatment is most often indicated for spasticity problems in a limited number of muscles, both in children with unilateral and bilateral spasticity. SDR is a neurosurgical procedure that involves partial sensory deafferentation at the lumbar and first sacral nerve rootlets. This procedure results in permanent reduction of muscle tone in the lower limbs [6]. SDR is most often used for people with walking ability and generally high spasticity level in both lower extremities. ITB is a continuous administration of baclofen into the intrathecal space from an implanted pump and through a catheter entering the spinal canal. Baclofen reduces the increased muscle tone from spasticity and/or dystonia, by binding to GABA-receptors and blocking excitatory neurotransmitters [7]. The baclofen dosage can be adjusted by telemetry. ITB is most often used for people with severe gross motor impairment with a generally high spasticity level.

*CP-North: Living life with cerebral palsy in the Nordic countries?* is a multinational research program where medical, health economics, public health and social outcomes associated with living with CP, for the individuals and their caregivers, are being investigated in Denmark, Finland, Iceland, Norway and Sweden. The CP-North data were extracted from each of the follow-up programs for individuals with CP in Denmark, Iceland, Norway and Sweden. These data are also linked to multiple national health registers in the aforementioned countries [8]. Finland does not have a national follow-up program for CP and is therefore represented with a cohort from southern Finland, an area comprising 30% of the Finnish population. Iceland’s follow-up program is not national per se, as it does cover the majority of the capital area. While Scotland is not part of the CP-North program, it was included in this study because they have a national follow-up program for CP similar to Sweden, Norway, Denmark and Iceland and the opportunity to submit corresponding data.

The aim of this study was to describe and compare the use of SDR, ITB and BTX-A treatments in children and adolescents with CP born from 2002 to 2017 in Denmark, Finland, Iceland, Norway, Scotland and Sweden as part of the CP-North research program. Our hypothesis was that there are differences in the distribution of spasticity treatment between the countries due to different access to the treatment methods and/or divergences in preferences of treatment methods among professionals across countries. Differences may also be due to possible differences in the distribution of CP subtypes and GMFCS levels.

## Methods

This is a registry-based study using data from the national follow-up programs for individuals with CP in Denmark, Iceland, Norway, Scotland, Sweden, and a defined cohort in Finland. Through the national follow-up programs, data are collected on fine and gross motor function, joint range of motion, degree of spasticity, use of assistive devices, physical and occupational therapy, physical activity in kindergarten/school and leisure time, imaging and treatments, including the three spasticity reducing methods, among others. Data are collected once or twice per year, or every other year depending on the child/adolescent’s gross motor function level and age [9, 10].

The Swedish Follow-Up Program for CP (CPUP) was established in 1994 in southern Sweden and expanded over time to include the entire country by 2005. The CPUP covers more than 95% of individuals with CP born 2000 or later [11]. The Norwegian CP Follow-Up Program was established in 2006 in one of four health care regions (southeastern health region) comprising 57% of children born 2002 to 2005, and was expanded nationally in 2010 to include all children and adolescents with CP born 2006 and later with a coverage of more than 90% of the population [12]. The Danish CP Follow-Up Program (CPOP) was established in southern Denmark in 2010 in one of five health care regions, and included children born 2000 and later. The Danish CPOP was expanded nationally to include all five regions during 2015–2018 for children born 2008 and later, with the exception of the northern Denmark region, which included children born 2007 and later. Coverage in the various birth cohorts/regions in Denmark is estimated to be 93% [13]. The Icelandic CP Follow-Up Program (CPEF) was established in 2012 and mainly includes children and adults with CP in the Reykjavik area. Based on an estimated prevalence of two per 1,000 live births, it is estimated that the program covers approximately 58% of the children and adolescents with CP in Iceland. The Scottish CP Follow-Up Program (CPIPS) was established nationally in 2013 and covers > 95% of individuals with CP born 2002 and later [14]. The Finnish cohort represents individuals born 2002–2017 in southern Finland and the Helsinki University Hospital catchment area, covering 1.7 million of Finland’s 5.5 million inhabitants. The proportion of individuals in the cohort in relation to the estimated total number of individuals with CP in the region is estimated at 68% (*n* = 442). However, all individuals with CP in the area treated with the three spasticity treatment methods of interest are included in the cohort. To avoid inflation of the estimates, some of the calculations are therefore performed with an estimated total population of CP based on a prevalence of two per 1,000 live births (*n* = 654) [15].

Aggregate data were collected from each CP-North follow-up program for children born from 2002 to 2017 considering their age, sex, CP subtype, gross motor function and spasticity treatments (SDR, ITB and BTX-A in the lower extremities). The Finish cohort data were extracted from medical records. Gross motor function was classified according to the 1997 Gross Motor Function Classification System (GMFCS) [16] or the GMFCS Expanded & Revised version from 2007 [17]. The dates in which the spasticity treatments were performed were available in all programs/cohort with the exception of when SDR and ITB were performed in Scotland, which was not available before 2014.

### Statistical analysis

Descriptive statistics were performed using means and standard deviations (SD) for continuous variables and absolute numbers and percentages for categorical and ordinal variables. Differences in proportions of treatment type (SDR, ITB, BTX-A) were analyzed by country, sex, age at treatment and GMFCS level. Statistical significant differences were analyzed using Pearson chi-square tests for categorical variables (country, sex, GMFCS level) and one-way ANOVA for the continuous variable (age). To reduce the risk of family wise type I error due to multiple comparisons on the same sample, the Bonferroni correction was applied to the four omnibus tests. If *p* ≤ 0.01 on the omnibus tests, pairwise comparisons were performed. Ninety-five percent confidence intervals (CI) are reported when relevant. Differences in treatments over time are presented descriptively.

SPSS version 25 was used for all analyses.

## Results

A total of 8,817 individuals with CP born between 2002 and 2017 were included. Of these, 5,093 (57.8%) were boys. Distributions according to country, sex, birth year, and GMFCS levels are presented in Table 1. In total, 142 individuals with CP (1.6%) were treated with SDR and 261 (3.0%) with ITB (Table 2). In the most recent 2017–2018 reports of data to the registers, 1,257 of 7,729 individuals (16.3%) were reported as having been treated with BTX-A in the lower extremities since the previous report (Table 2).

**Table 1:** Characteristics of children with cerebral palsy (CP)

	**Sweden**		**Norway**		**Denmark**		**Iceland**		**Scotland**		**Finland**	**Total**	
	Total	Reports 2017–18	Total	Reports 2017–18	Total	Reports 2017–18	Total	Reports 2017–18	Total	Reports 2017–18	Total	Total	Reports 2017–18
**Sex. n (%)**													
** Boys**	2,166 (57.9)	1,981 (58.5)	878 (57.7)	803 (57.9)	749 (58.6)	691 (58.5)	43 (51.8)	39 (55.9)	1,006 (57.2)	718 (56.5)	251 (56.8)	5,093 (57.8)	4,483 (58.0)
** Girls**	1,575 (42.1)	1,407 (41.5)	644 (42.3)	584 (42.1)	530 (41.4)	490 (41.5)	39 (48.2)	29 (43.3)	745 (42.5)	545 (43.2)	191 (43.2)	3,724 (42.2)	3,246 (42.0)
**Birth year, n (%)**													
** 2002–03**	566 (15.1)	474 (14.0)	164 (10.8)	139 (10.0)	56 (4.4)	42 (3.6)	8 (9.6)	6 (9.0)	210 (12.0)	136 (10.8)	52 (11.8)	1,056 (12.0)	849 (11.0)
** 2004–05**	499 (13.3)	429 (12.7)	177 (11.6)	158 (11.4)	54 (4.2)	46 (3.9)	18 (21.7)	13 (19.4)	256 (14.6)	170 (13.5)	49 (11.1)	1,053 (11.9)	865 (11.2)
** 2006–07**	573 (15.3)	505 (14.9)	276 (18.1)	244 (17.6)	93 (7.3)	81 (6.9)	12 (14.5)	9 (11.9)	263 (15.0)	179 (14.2)	54 (12.2)	1,271 (14.4)	1,072 (13.9)
** 2008–09**	549 (14.7)	511 (15.1)	272 (17.9)	244 (17.6)	270 (21.1)	248 (21.0)	9 (10.8)	8 (11.9)	332 (19.0)	228 (18.1)	61 (13.8)	1,493 (16.9)	1,300 (16.8)
** 2010–11**	538 (14.4)	514 (15.2)	225 (14.8)	211 (15.2)	265 (20.7)	244 (20.7)	19 (22.9)	17 (25.4)	254 (14.5)	190 (15.0)	67 (15.2)	1,368 (15.5)	1,243 (16.1)
** 2012–13**	453 (12.1)	427 (12.6)	195 (12.8)	189 (13.6)	226 (17.7)	209 (17.7)	10 (13.3)	9 (13.4)	231 (13.2)	183 (14.5)	69 (15.6)	1,184 (13.4)	1,086 (14.1)
** 2014–15**	386 (10.3)	369 (10.9)	135 (8.9)	128 (9.2)	196 (15.3)	192 (16.3)	5 (6.0)	5 (7.5)	161 (9.2)	137 (10.8)	55 (12.4)	938 (10.6)	886 (11.5)
** 2016–17**	177 (4.7)	159 (4.7)	78 (5.1)	74 (5.3)	119 (9.3)	119 (10.1)	1 (1.2)	1 (1.5)	44 (2.5)	40 (3.2)	35 (7.9)	454 (5.1)	428 (5.5)
** Total**	3,741 (100)	3,388	1,522 (100)	1,387	1,379 (100)	1,181	88 (100)	68	1,751 (100)	1,263	442 (100)	8,817 (100)	7,729 (100)
**GMFCS levels, n (%)**													
** I**	1,659 (44.3)	1,508 (44.5)	805 (52.9)	715 (51.6)	657 (51.4)	601 (50.9)	33 (42.2)	25 (36.8)	668 (38.0)	413 (32.5)	189 (42.8)	4,011 (45.5)	3,451 (45.8)
** II**	580 (15.5)	516 (15.2)	255 (16.8)	238 (17.2)	204 (15.9)	197 (16.7)	18 (21.7)	17 (25.0)	355 (20.2)	238 (18.7)	97 (21.9)	1,509 (17.1)	1,303 (16.9)
** III**	329 (8.8)	318 (9.4)	103 (6.8)	98 (7.1)	84 (6.6)	84 (7.1)	10 (12.0)	9 (13.2)	162 (9.2)	150 (11.8)	39 (8.8)	727 (8.2)	698 (9.0)
** IV**	518 (13.8)	494 (14.6)	141 (9.3)	134 (9.7)	137 (10.7)	132 (11.2)	14 (15.7)	13(17.6)	233 (13.2)	199 (15.7)	55 (12.4)	1,098 (12.4)	1,027 (13.3)
** V**	598 (16.0)	552 (16.3)	203 (13.3)	192 (13.8)	195 (15.2)	165 (13.7)	4 (6.0)	4 (7.4)	341 (19.4)	270 (21.3)	56 (12.7)	1,397 (15.9)	1,239 (16.0)
** Unclassified**	57 (1.5)	0 (0)	15 (1.0)	10 (0.7)	2 (0.2)	2 (0.2)	0 (0)	(0)	0 (0)	0 (0)	6 (1.4)	80 (0.9)	18 (0.2)

**Table 2:** Number of treatments and mean age at treatment by country with standard deviation (SD) and 95% confidence intervals (CI)

	**SDR**			**ITB**			**BTX-A**		
	n	Mean age (SD)	95% CI	n	Mean age (SD)	95% CI	n	Mean age (SD)	95% CI
Sweden	45	5.04 (1.78)	4.51–5.58	84	7.42 (2.98)	6.77–8.06	646	9.16 (3.72)	8.88–9.45
Norway	19	4.53 (1.81)	3.66–5.40	56	6.32 (3.02)	5.51–7.13	280	8.19 (3.12)	7.82–8.55
Denmark	19	7.26 (3.67)	5.50–9.03	47	7.62 (3.51)	6.59–8.65	101	7.09 (2.81)	6.53–7.64
Iceland	0			< 5			11	10.27 (2.15)	8.83–11.72
Scotland^a^	59			62			180	8.89 (3.60)	8.36–9.42
Finland	0			11	10.09 (3.30)	7.87–12.31	39	9.95 (2.84)	9.03–10.87

^a^ Age at SDR and ITB not available for Scotland

## Spasticity treatments by country

**Fig. 1:**
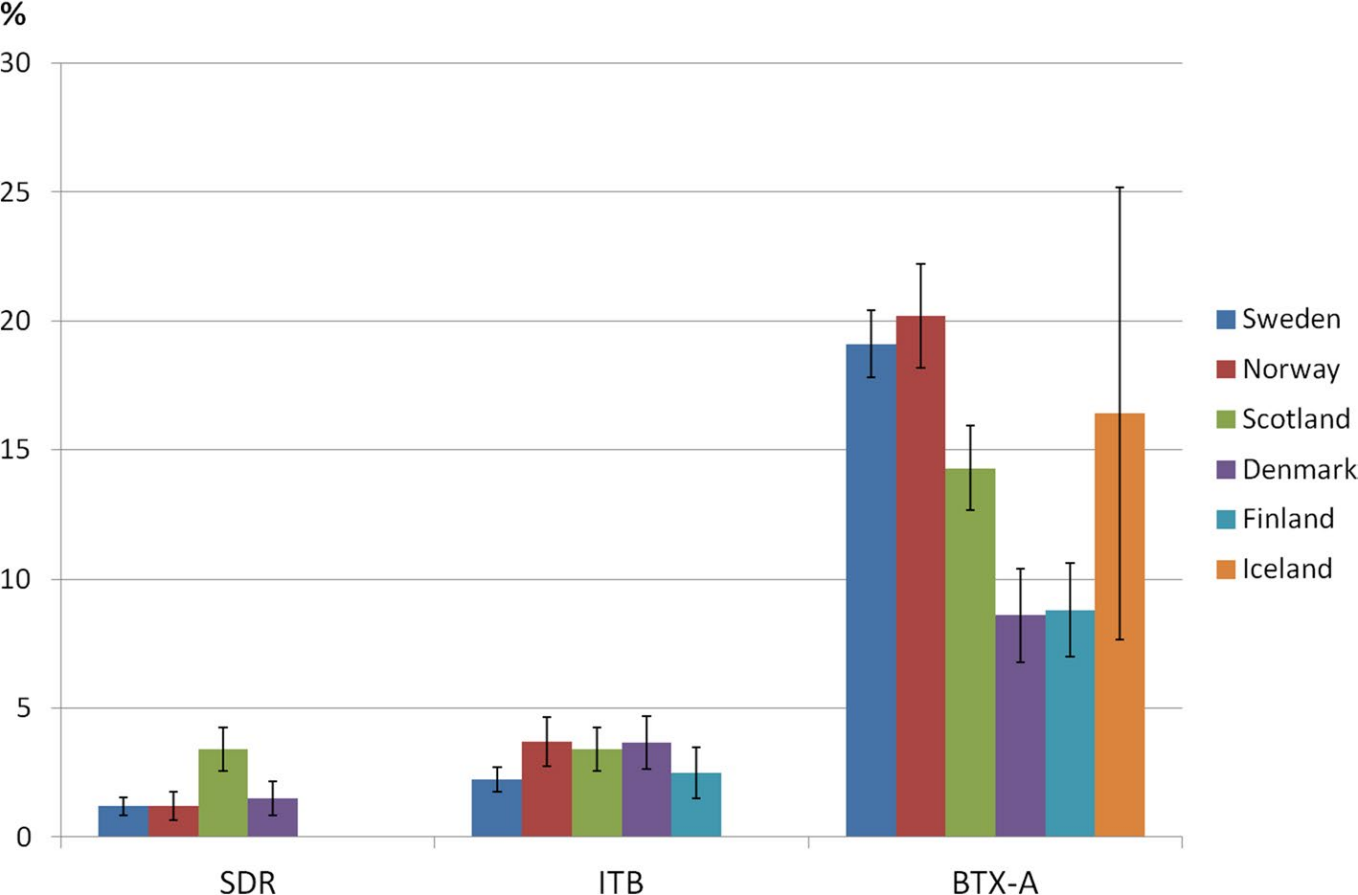
Proportions (%) of children treated with selective dorsal rhizotomy (SDR), intrathecal baclofen pump (ITB) and botulinum toxin-A (BTX-A) with 95% confidence intervals in Sweden, Norway, Scotland, Denmark, Finland and Iceland. < 5 individuals treated with ITB in Iceland

The distributions and mean ages of children who have undergone each spasticity treatment (SDR, ITB, BTX-A) per country are shown in Table 2, and proportions with 95% CIs in Fig. 1.

### SDR

SDR was used in Sweden, Norway, Denmark and Scotland, but not in Iceland or Finland. Overall, there were statistically significant differences among countries, χ^2^ = 36.68, df = 3, *p* < 0.01. Pairwise comparisons revealed that Scotland (3.4%) performed more SDR surgeries than Sweden (1.2%), χ^2^ = 30.14, df = 1, Norway (1.2%), χ^2^ = 15.75, df = 1 and Denmark (1.5%), χ^2^ = 10.46, df = 1, *p* < 0.01 for all comparisons. There were no statistically significant differences on SDR between Sweden, Norway and Denmark.

### ITB

All countries used ITB. Overall, there were statistically significant differences among the countries, χ^2^ = 13.98, df = 4, *p* < 0.01. Pairwise comparisons revealed that Sweden (2.2%) performed statistically significantly fewer ITBs than Norway (3.7%), χ^2^ = 8.59, df = 1, Denmark (3.7%), χ^2^ = 7.66, df = 1 and Scotland (3.5%), χ^2^ = 7.74, df = 1, *p* < 0.01 for all comparisons. There were no statistical differences in the use of ITB between Sweden and Finland (2.5%), or between Norway, Denmark and Scotland. Calculated with the estimated total population of children in the Helsinki area (*n* = 654) the proportion of children treated was 1.7%. Due to small numbers, data on ITB are not reported for Iceland.

### BTX-A

All countries used BTX-A in the lower extremities during the latest 2017–2018 reporting period. There were overall statistically significant differences among the countries, χ^2^ = 108.52, df = 5, *p* < 0.01. Norway (20.2%) and Sweden (19.1%) were statistically significantly more likely to have used BTX-A since the last assessment than Denmark (8.6%), (Norway/Denmark χ^2^ = 68.35, df = 1, Sweden/Denmark χ^2^ = 70.80, df = 1) Finland (8.8%) (Norway/Finland χ^2^ = 30.06, df = 1, Sweden/Finland χ^2^ = 27,94, df = 1) and Scotland (16,3%) (Norway/Scotland χ^2^ = 16.24, df = 1, Sweden/Scotland χ^2^ = 14.61, df = 1), all *p* < 0.01. Scotland was statistically significantly more likely to have used BTX-A since the last report than Finland and Denmark (Scotland/Finland χ^2^ = 8.62, df = 1, Scotland/Denmark χ^2^ = 19.49, df = 1) both *p* < 0.01. There were no statistically significant differences in BTX-A use since last report between Norway and Sweden, between Denmark and Finland or between Iceland and the other countries. Calculated with the estimated total population of children in the Helsinki area (*n* = 654) the proportion of children treated was 6.0%.

## Spasticity treatments by sex and country

**Fig. 2:**
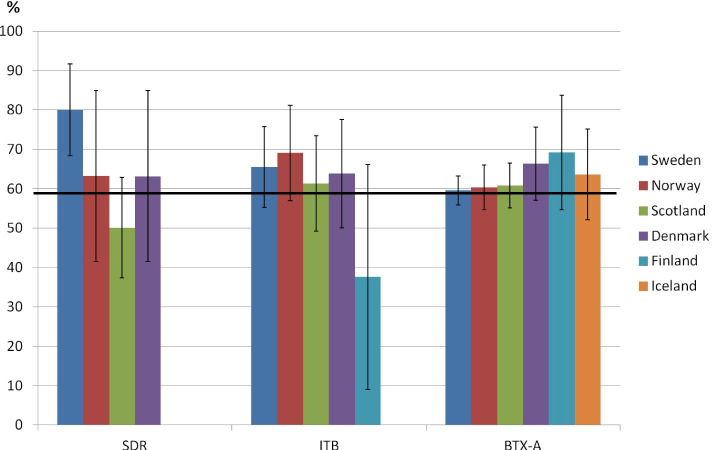
Proportions (%) of boys treated with selective dorsal rhizotomy (SDR), intrathecal baclofen pump (ITB) and botulinum toxin-A (BTX-A) with 95% confidence intervals in the Sweden, Norway, Scotland, Denmark, Finland and Iceland. Line marks proportion boys in the total material (58%)

There were no overall significant differences between sexes per spasticity treatment among the countries. Proportions of boys per spasticity treatment among the countries are shown in Fig. 2.

### SDR

In total, of the 142 children who had received SDR 90 (63.4%) were boys. The Pearson chi-square test for sex differences on SDR by country was not significant, χ2 = 9.35, df = 3, *p* = 0.03 and therefore pairwise comparisons were not reported. Of interest, however, 80% of the children who had an SDR in Sweden were boys compared to 51% in Scotland.

### ITB

In total, 161 (64.7%) of the 249 children who had undergone ITB were boys. The Pearson chi-square test for sex differences on ITB by country was not significant, χ^2^ = 0.60, df = 3, *p* = 0.89 and therefore pairwise comparisons were not reported.

### BTX-A

There were no significant differences in the proportion of boys who had BTX-A in the lower extremities among the countries; Sweden (60.2%), Norway (60.4%), Denmark (66.3%), Finland (69.2%), Iceland (63.6%), Scotland (61.1%). The Pearson chi-square test for sex differences on BTX-A by country was, χ^2^ = 2.56, df = 5, *p* = 0.77 and therefore pairwise comparisons were not reported.

## Spasticity treatments by age and country

### SDR

The mean age at the time of SDR surgery was available in Sweden, Norway and Denmark. There were overall statistically significant differences between group means as determined by one-way ANOVA (*F*(2, 80) = 7.82), *p* < 0.01. Post-hoc comparisons revealed that mean age of SDR was statistically significantly higher in Denmark (7.3 years, SD = 3.66) compared to Sweden (5.0 years, SD = 1.78) and Norway (4.5 years, SD = 1.81), both *p* < 0.01 (Table 2). There was no statistically significant difference in mean age between Sweden and Norway.

### ITB

There were overall statistically significant differences between group means as determined by one-way ANOVA (*F*(3, 194) = 4.91), *p* < 0.01. The mean age at ITB surgery was higher in Finland (10.1 years, SD = 3.30) compared to Denmark (7.6, SD = 3.51, *p* < 0.05), Sweden (7.4 years, SD = 2.98, *p* < 0.01) and Norway (6.3 years, SD = 3.02, *p* < 0.01). Difference in mean age between Norway and Denmark was also statistically significantly different (*p* < 0.05) (Table 2). Due to small cell sizes, data on ITB are not reported for Iceland.

### BTX-A

There were overall statistically significant differences between group means as determined by one-way ANOVA (*F*(5, 1251) = 9.33), *p* < 0.01. The mean age for children treated was statistically significantly lower in Denmark (7.1 years, SD = 2.81) than in Sweden (9.2 years, SD = 3.72), Norway (8.2 years, SD = 3.12), Finland (10.0, SD = 2.84), Iceland (10.3 years, SD = 2.15) and Scotland (8.9 years, SD = 3.60), *p* < 0.01 for all. The mean age was also statistically significantly lower in Norway compared to Sweden (*p* < 0.01) and Finland (*p* < 0.01) (Table 2).

## Spasticity treatments by GMFCS level and country

**Fig. 3:**
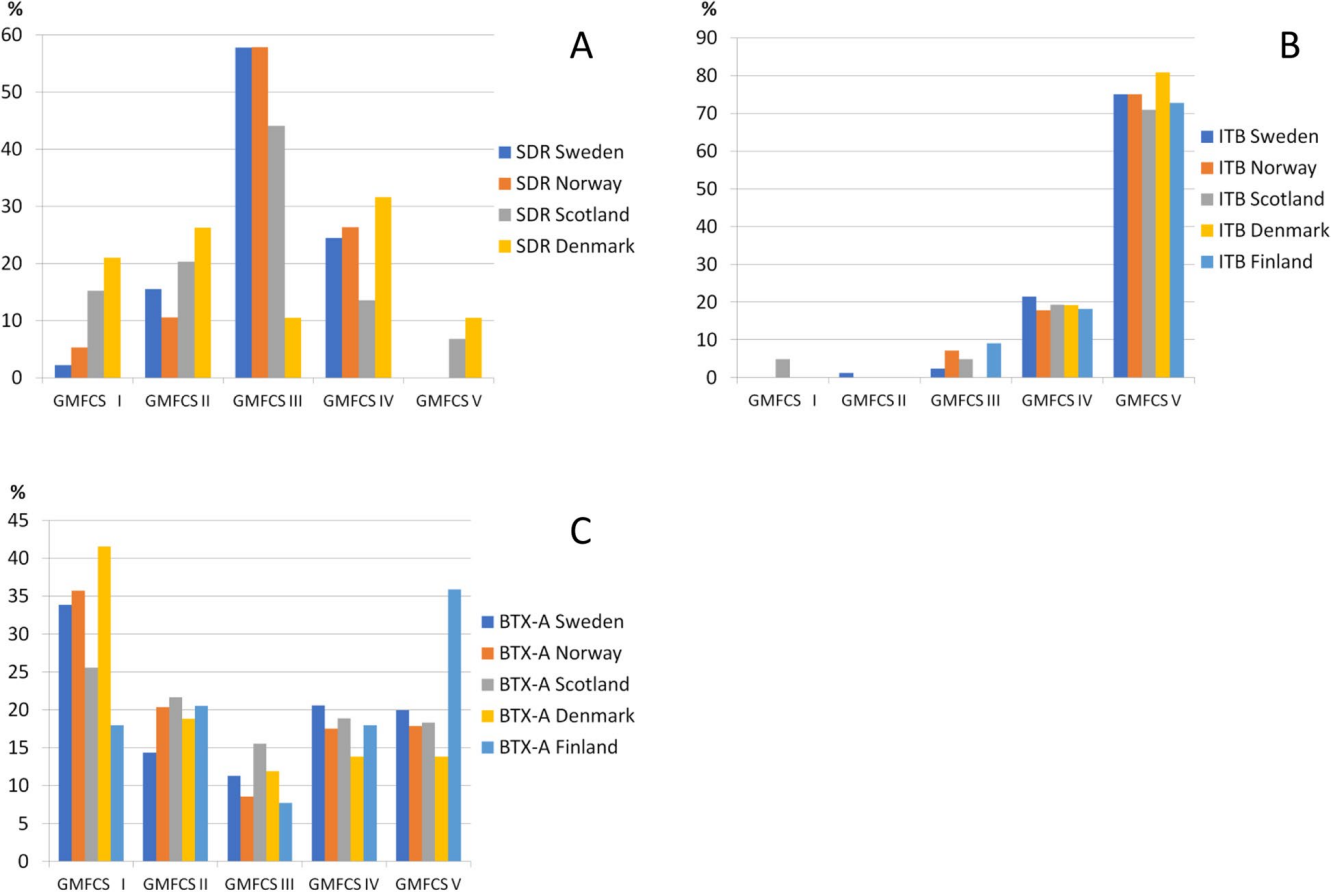
Proportions (%) of children treated with **a** selective dorsal rhizotomy (SDR), **b** intrathecal baclofen pump (ITB) and **c** Botulinum toxin-A (BTX-A) related to Gross Motor Function Classification System (GMFCS) level in Sweden, Norway, Denmark, Scotland and Finland. No child treated with SDR and < 5 treated with ITB in Iceland

These results are presented descriptively. No significance tests were performed due to small counts and the vast number of tests that would be required. The proportion of children per spasticity treatment and GMFCS level among the countries are shown in Fig. 3.

### SDR

Treatment with SDR was most common in GMFCS level III in Norway (57.9%), Sweden (57.8%) and Scotland (44.1%), and among the least common in Denmark (10.5%). Children in the least common GMFCS levels in Sweden and Norway to be treated with SDR were in GMFCS levels I (2.2 and 5.3% respectively) and V (0%), and levels IV and V in Scotland (13.6 and 6.8% respectively).

### ITB

ITB treatment was most common in GMFCS level V in all countries, ranging from 71.0% in Scotland to 80.9% in Denmark. Due to small cell sizes, data on ITB are not reported for Iceland.

### BTX-A

While BTX-A treatment was generally performed in all GMFCS levels, it was most commonly performed in GMFCS level I in Sweden (33.8%), Norway (35.7%) and Denmark (41.6%). Conversely, in Scotland, Finland and Iceland, fewer individuals were treated in GFMCS level I (25.6, 17.9 and 9.1% respectively). In Finland, most individuals were treated in GMFCS level V (35.9%).

**Fig. 4:**
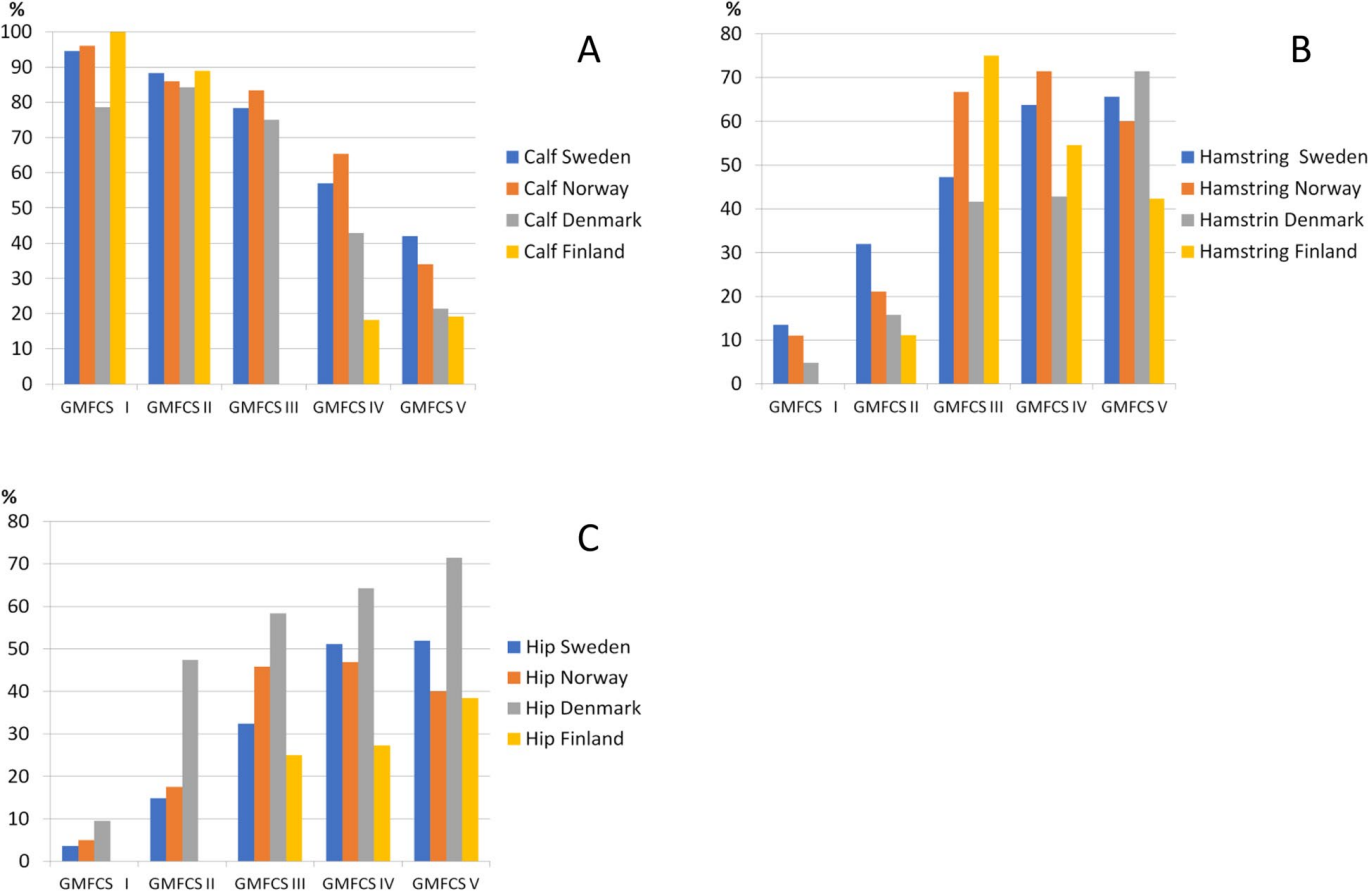
Proportion of Botulinum toxin-A (BTX-A) treatment (%) in **a** calf-muscles, **b** hamstrings and **c** hip muscles in different Gross Motor Function Classification System (GMFCS) levels among the children treated with BTX-A in Sweden, Norway, Denmark and Finland

The lower extremity muscles that were treated with BTX-A were analyzed for Sweden, Norway, Denmark and Finland (Fig. 4). This information was not registered in Scotland and there were too few participants in Iceland to ethically report on this. The most common muscle treated with BTX-A in all countries was the calf muscle, with the highest proportion in GMFCS level I (94–100%), except for Denmark where the highest proportion was in GMFCS level II (84%). Treatment with BTX-A in the calf muscle decreased by GMFCS-levels to 19–42% in level V. BTX-A treatment of hamstring muscles increased by GMFCS levels to level IV in Norway (71.4%) and Finland (54.5%) and to level V in Sweden (66%) and Denmark (71%). Treatment of hip muscles increased to level IV in Norway (47%) and to level V in Sweden (52%), Denmark (71%) and Finland (38.4%) (Fig. 4).

## Spasticity treatments over time by country

### SDR

**Fig. 5:**
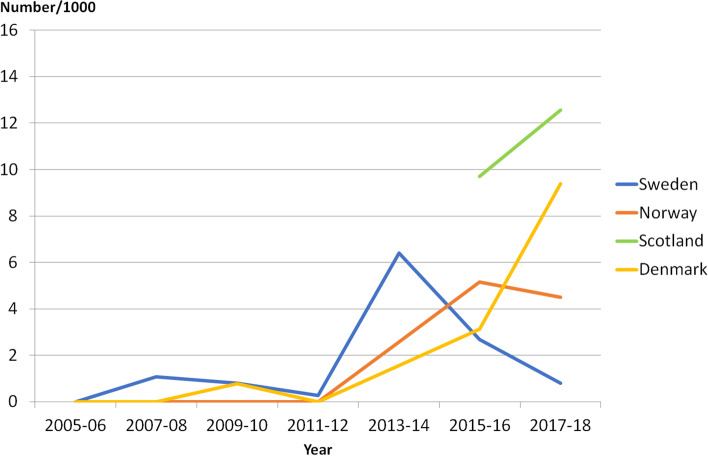
Number of children treated with Selective Dorsal Rhizotomy (SDR) in Sweden, Norway and Denmark per 1000 participants in each country's cohort during the years 2005—2018. No child treated in Finland or Iceland. Information on year of treatment before 2015 not available for Scotland

The proportion of individuals treated with SDR per year increased in Sweden until 2013–2014, in Norway until 2015–2016, and in Denmark until 2017–18. Thereafter, the number of SDR surgeries decreased in Sweden and remained stable in Norway (Fig. 5). In Scotland, the total number who had received this treatment was recorded at the commencement of the CPIPS program in 2014, but their age at time of surgery was not.

### ITB

**Fig. 6:**
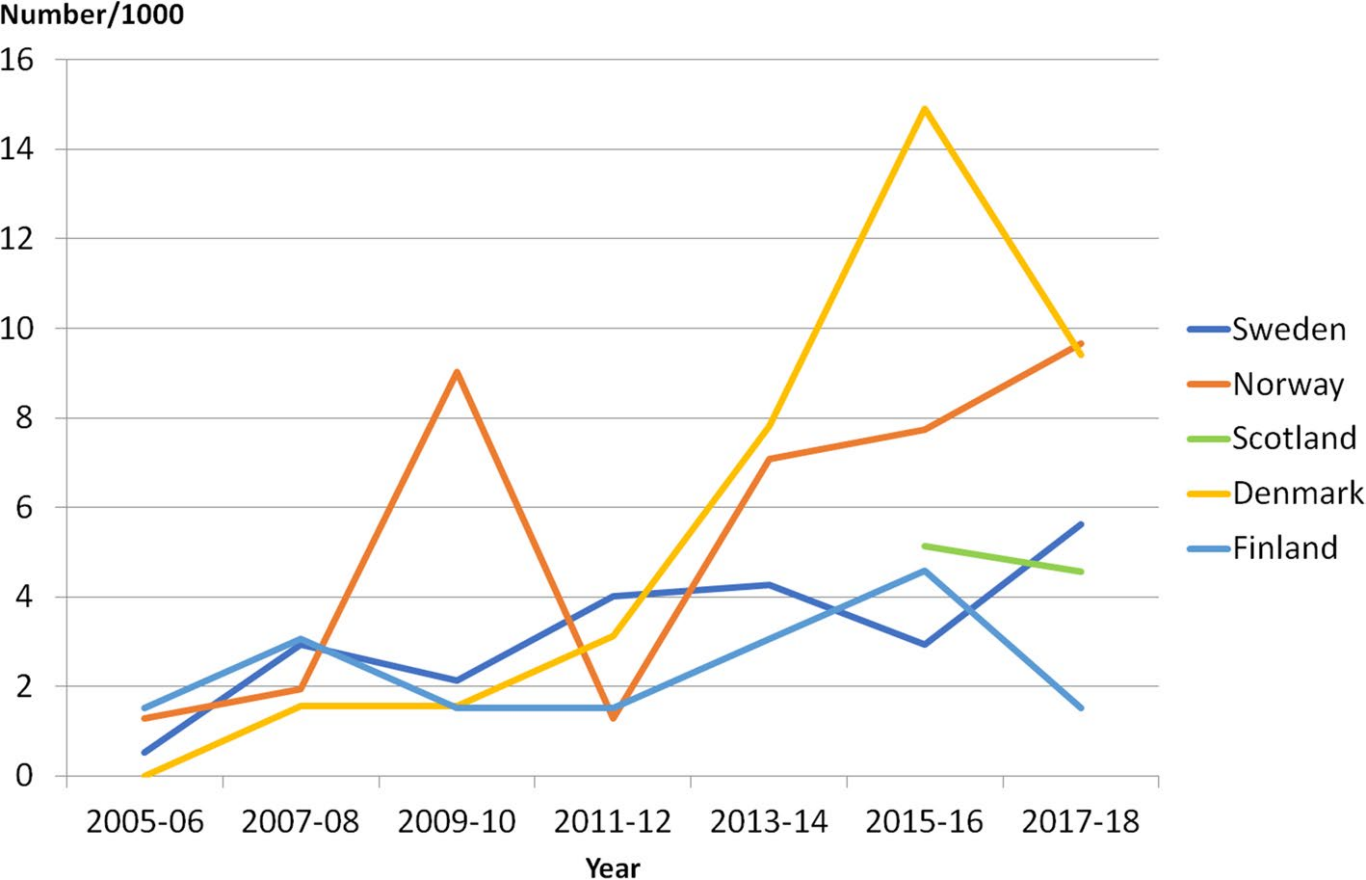
Number of children treated with intrathecal baclofen pump (ITB) in Sweden, Norway, Scotland and Finland per 1,000 participants in each country’s cohort during the years 2005–2018. Less than five children treated in Iceland. Result from Finland based on estimated total population (*N* = 654). Information on year of treatment before 2015 not available for Scotland

Treatment with ITB increased in Denmark until 2015–2016 followed by a slight decrease. In Sweden, Norway and Finland, the number of ITB surgeries per year has varied without any clear trends (Fig. 6).

### BTX-A

Data on use of BTX-A over time was not available and therefore could not be analyzed.

## Discussion

This study showed statistically significant differences among six northern European countries regarding the proportion of children with CP treated with three spasticity-reducing methods and mean age of treatment. Differences in treatment methods related to GMFCS level and the use of the treatment methods over time were presented descriptively. There are no age-related differences regarding insurances or national regulations for treatment between the countries.

A previous CP-North study showed that the proportion of children with spastic bilateral CP was similar between the countries, while spastic unilateral CP was more frequent in Norway [1]. SDR and ITB treatment are only given to people with bilateral spasticity but the difference in unilateral CP may have affected the results regarding BTX-A treatment. Differences in GMFCS distribution between countries could also affect the results, but the analysis of treatment related to GMFCS level showed significant differences between countries. There was a lower proportion of children born 2002–2007 in Denmark. Despite this, Denmark has a higher average age for treatment with SDR and ITB, but a lower average age among children treated with BTX-A which could be explained by the difference in birth year distribution.

## Spasticity treatments by sex and country

None of the spasticity treatments differed statistically significantly by sex across the countries. Previous research on spasticity reducing methods in Sweden have reported sex differences. A Swedish population-based study showed that significantly more boys than girls were treated with SDR [18]. In another Swedish population-based study based on treatments reported 2014–2015, significantly more boys received BTX-A [18]. However, this difference was not found in reports from 2016–2017 [19], or in the present study. Himmelmann et al. studied access to ITB in Europe and found that more boys than girls were treated with ITB [20]. The study included 116 individuals born 1990–2005 and evaluated in 2011 and 119 individuals born 1999–2015 and evaluated in 2019. To the best of our knowledge, there are no published sex differences in degree of spasticity or gross motor function that warrant differences in spasticity reducing treatment.

### SDR

The proportions of individuals treated with SDR ranged from zero in Finland and Iceland to 3% in Scotland. Sweden, Norway and Scotland had the same distribution with respect to GMFCS level, with most children operated in GMFCS level III, while Denmark had a reverse distribution with the least proportion of children operated in level III. There was also a difference in the number of children treated with SDR over time. SDR operations have decreased in Sweden and increased or remained stable in Norway and Denmark. SDR is no longer performed in Finland. This decision was based on the results of a comparison of 21 children who underwent SDR in 1991–1998 and 21 who received physiotherapy alone [21]. The study revealed no significant difference in motor function between the two groups. The differences between countries may also be due to the availability of the operation. In Sweden, SDR has been performed since 1993, whereas in Norway SDR operations became available in late 2019. Previously, Norwegian children with CP received this treatment abroad paid by the state. In Scotland, an SDR program was established in 2018 allowing access to a national multidisciplinary review and decision-making teams, with surgery now available in two of the main children’s hospitals. Prior to this, most SDR operations in Scotland were performed abroad, often at the request of the families without formal assessment by local teams. In Denmark, SDRs have been performed at one hospital since 1992, but a number of families have chosen to have the operation performed abroad, often at their own expense. This could help explain the different GMFCS distribution and higher mean age at surgery of the children in Denmark who have undergone SDR.

Several studies have shown positive short-term results after SDR, while there are differing opinions as to whether the surgery provides lasting benefits [22]. This may also explain the large differences in treatment frequency between countries. It should be noted that some of these spasticity treatments are somewhat controversial among professionals and families of children with CP. This is particularly true for SDR and BTX-A. This may influence what treatment professionals recommend and what families are inclined to accept as treatment for their children and may differ both within and across countries. There are also likely trends among the families of children with CP within each country with certain treatments being in favour at certain times. As a consequence, trends in treatment methods likely varies over time, with different modalities of treatment being popular in each country at any given time. External influences by clinics abroad promoting and offering treatments, for example SDR, are likely to have had an effect. This is probably the case in the current generation of parents who use social media, where there is a continual access to instant information.

### ITB

The proportions of individuals treated with ITB were higher in Norway, Scotland and Denmark compared with Sweden. The differences between Sweden, Norway and Scotland are most evident in recent years. All countries in this study used ITB primarily in individuals in GMFCS levels IV-V. Improved gait capacity with ITB treatment has also been reported for ambulant children [23]. However, only ten individuals in this study in GMFCS level I-II had been treated with ITB. This is likely due to SDR being used as the more common treatment modality to improve gait in children with CP who have spasticity.

### BTX-A

There were large differences, ranging from seven to 20%, in proportions of children treated with BTX-A, with the highest number treated in Sweden and Norway. However, there are also large regional differences regarding use of BTX-A treatment reported from Norway and Sweden [24, 25]. Follow-up programs for CP have existed the longest in Sweden and Norway, and the continuous monitoring of muscle tone means that indications for BTX-A treatment are recognized more regularly, and thus it was used more often. BTX-A is provided free of charge for the families in all six countries. In Finland, fewer children in GMFCS level I and more children in GMFCS V were treated with BTX-A compared to the other countries. Our hypothesis is that this difference can be at least partially explained by the fact that Finland does not use a standardized follow-up program. Differences in use of BTX-A may also be explained by access to treatment and local practices, as well as utilization of other treatments such as transcutaneous neuromuscular electrical stimulation [26] This indicates that the differences are more related to professional discussion/disagreement and parental preferences than to accessibility. The muscles being treated were quite similar in all countries.

## Strengths and limitations

A strength of this study is that the CP cohorts from Sweden, Norway, Denmark, and Scotland represent more than 90% of the total population in each country, and therefore reduce selection bias and increase external validity. However, during the years 2002–2004, some of the registers were still in the process of being implemented and enrolling participants, which may have affected the results related to these years. While the cohorts from Iceland and Finland are not national, the distributions of GMFCS and sex are similar to those of the other countries. However, this does not rule out a possible selection bias related to the total population in these countries. We did not have data to analyze the development of BTX-A treatment over time as for ITB and SDR. The proportion of individuals reported in 2017–2018 for follow-up of BTX-A represented 86% of the total cohorts. Here, too, there may be a selection bias, but the distribution by sex, GMFCS level and year of birth were similar between the individuals reported 2017–2018 and the total cohort in each country.

## Conclusion

The purpose of this study was to compare differences and similarities among the three treatment options for spasticity across six northern European countries. In summary, there were statistically significant differences between the countries in the use of the three spasticity reduction methods. This may be due to differences in the availability of these methods and/or different indications and local practices and preferences and not necessarily represents what improves the spasticity the most. However, the optimal indications for these treatments remain to be investigated. These CP registers give us the opportunity to study and compare the long-term results with the different treatment methods and unveil possibilities for further studies on other outcomes of the interventions. It is also possible to create comparison groups of children with similar untreated symptoms, and the big dataset gives us opportunities for subgroup analyses, such as children in the different GMFCS levels.

## Data Availability

The datasets used and/or analyzed during the current study are available from the corresponding author on reasonable request.

## Abbreviations

ANOVA: Analysis of variance
BTX-A: Botulinum toxin-A
CI: Confidence Interval
CP: Cerebral Palsy
CPUP: Cerebral Palsy Follow-Up Program in Sweden
CPOP: Cerebral Palsy Follow-Up Program in Denmark
CPEF: Cerebral Palsy Follow-Up Program in Iceland
CPIPS: Cerebral Palsy Follow-Up Program in Scotland
GABA: Gamma-aminobutyric acid
GMFCS: Gross Motor Function Classification System
ITB: Intrathecal baclofen pump therapy
SD: Standard deviation
SDR: Selective dorsal rhizotomy
